# A Comparative Study of Patients Undergoing Tonsillectomy Using the Cold Steel Dissection Method and Electrocautery Technique

**DOI:** 10.7759/cureus.79724

**Published:** 2025-02-26

**Authors:** Shashikant N Dorkar, Sachin S Nilakhe, Shahana A L

**Affiliations:** 1 Otolaryngology, Bharati Vidyapeeth (Deemed to be University) Sangli, Sangli, IND

**Keywords:** cold steel dissection method, complications of tonsillectomy, duration of surgery, electrocautery technique, tonsillectomy

## Abstract

Aim

The aim of this study was to compare the outcomes and complications in patients undergoing tonsillectomy using the cold steel dissection method and electrocautery technique.

Materials and methods

A prospective observational study was conducted including 350 patients undergoing tonsillectomy in the ENT Department of a tertiary care hospital who met specific inclusion and exclusion criteria. Data of patients were collected using a predesigned proforma. Intraoperative outcomes such as the duration of surgery (in minutes) and intraoperative blood loss (in milliliters) were measured, and preoperative and postoperative outcomes such as pain scores at 24 hours and at day 3 and postoperative hemorrhage were also measured. These parameters were analyzed to compare the efficacy and safety of tonsillectomy using the conventional cold steel dissection method and electrocautery technique.

Results

Among the 350 patients, 170 were male, with a mean age of 17.66 years. A comparison of study variables between the two groups showed a statistically significant (P< 0.05) difference in the duration of surgery, intraoperative blood loss, and 24 hours and day 3 postoperative pain scores for the cold steel dissection method and electrocautery method. Only two patients experienced bleeding complications after surgery, and the incidence of postoperative hemorrhage was not significantly different between the two techniques.

Conclusion

In our study, tonsillectomy using the electrocautery technique was advantageous in terms of shorter postoperative recovery time and reduced intraoperative blood loss. However, patients who underwent tonsillectomy using the electrocautery technique experienced slightly more postoperative pain compared to those who underwent the procedure using the cold steel dissection method.

## Introduction

Tonsillectomy is a surgical operation that involves the complete excision of the tonsil and its outer layer by carefully separating the tissue between the tonsil and its surrounding muscle wall. In some cases, the procedure may include the additional step of removing the adenoids, a process called adenoidectomy [1]. The tonsils are a component of Waldayer's ring, a collection of lymphoid tissue situated at the entrance of the respiratory and digestive tracts, specifically in the nasopharynx and oropharynx regions [2,3]. The tonsillectomy procedure is mainly performed for recurrent tonsillitis and obstructive sleep apnea. Even though tonsillectomy is often used to treat infections that keep coming back, most scientists think that the evidence is not strong enough to support its effectiveness [4]. For many years, the only notable and influential study on the topic was the one conducted by Paradise et al. and published in 1984, which remained a seminal work in the field until more recent research emerged [5]. Paradise et al. study's findings supported the effectiveness of tonsillectomy. The Paradise criteria, which define frequent sore throats as seven incidents in one year, five incidents per year for two years, or three incidents per year for three years, are still widely used today to determine eligibility for a tonsillectomy.

Research examining quality of life both before and after tonsillectomy has consistently shown that tonsil disease has a profoundly negative impact on overall well-being and that undergoing the surgery leads to substantial improvements in quality of life [6]. For decades, the traditional method of tonsillectomy has been the “cold steel” technique, which involves using metal instruments to remove the tonsils. This method has been widely used and is still commonly practiced today. There are many different ways to perform a tonsillectomy today, such as traditional cold dissection, monopolar and bipolar electrocautery, cryosurgery, ultrasonic scalpel, coblation excision, radiofrequency ablation, and laser-assisted removal [7]. This gives surgeons a lot of choices. Though various techniques exist, currently there is no final or precise evidence in the literature on the optimal method for performing tonsillectomy. No single technique has been universally accepted as the gold standard, as each method has its unique advantages and disadvantages. The choice of technique often depends on individual preferences and circumstances, highlighting the need for continued research and evaluation to determine the most effective approach.

The outcomes of tonsillectomy studies vary significantly, likely due to differences in factors such as patient demographics, sample size, environmental conditions, lifestyle, surgeon expertise, and study timing. The different results show that more research is needed and that individual factors need to be taken into account to reduce risks and improve patient outcomes. Following tonsillectomy, the most common complication is posttonsillectomy hemorrhage, accompanied by pain and other potential issues that require close monitoring and prompt management [8]. Tonsillectomy carries a significant risk, with a reported mortality rate of 1 in 1,100 to 1 in 1,600 cases, primarily due to bleeding complications during or after the procedure. Additionally, the surgery is associated with substantial morbidity, mainly due to hemorrhage and severe postoperative pain. Some patients may require hospital readmission for pain management and dehydration treatment, emphasizing the importance of close postoperative monitoring and effective pain management strategies to minimize these risks and ensure optimal patient outcomes.

This research evaluates and compares the outcomes and complications such as intraoperative blood loss and postoperative pain of two common tonsillectomy methods, cold steel dissection and electrocautery, in patients at a tertiary care center's otorhinolaryngology department. By looking at how well and safely these methods work, this study hopes to give surgeons and other healthcare professionals useful information that will help improve patient outcomes and care in the field of ear, nose, and throat (ENT) surgery.

## Materials and methods

A prospective observational study was conducted to compare outcomes and complications in patients undergoing tonsillectomy using the cold steel dissection method and electrocautery technique. The indoor patients in the Department of Otorhinolaryngology fulfilling the inclusion and exclusion criteria were included in the study. A total of 350 patients were considered for fulfilling the study criteria, and their data were collected and analyzed to compare the efficacy and safety of two surgical techniques. In the case of children, before enrollment in the study, caregivers were educated about the study's purpose, and written consent was taken. A standardized proforma was used to collect data. The proforma included a detailed medical history, clinical examination, and relevant investigations such as complete blood counts based on the child's history and examination findings. The patients were randomly assigned into two treatment categories in an alternating manner to avoid selection bias, ensuring that for every patient who underwent the cold steel dissection method, the next patient underwent the electrocautery technique using XcelLance Shalya TURoSeal Electrocautery Model No. TS12E02B-vbe (Navi Mumbai, Maharashtra, India).

Participants were eligible for inclusion if they met the following criteria: (i) diagnosed with chronic tonsillitis and are having a tonsillectomy using the cold steel dissection method and the electrocautery technique, (ii) in the age group of 10 years and above at the time of consent, and (iii) willing to participate in the study. Participants were excluded if they were diagnosed with clinical diagnoses other than chronic tonsillitis, such as tonsillar malignancy, known bleeding disorder, and tonsillolith.

The anesthesia team performed orotracheal or nasal intubation as per the institutional protocol. The patient was placed in Rose's position on the surgical table. A Boyle-Davis mouth gag with a tongue blade was inserted into the oral cavity and stabilized using Draffin's bipods. For adenotonsillectomy cases, the adenoid was first removed, followed by tonsillectomy using either the cold steel dissection method or the electrocautery technique. In the cold steel dissection method, extracapsular tonsillar dissection was performed, and bleeding was controlled by ligating the vessels with linen ties. The procedure was repeated on the other side. In the electrocautery technique, tonsillectomy was performed using electrocautery, and hemostasis was achieved with bipolar cautery. The surgeries were performed by different surgeons with experience of more than 20 years.

The study compared preoperative and postoperative outcomes based on various study variables. The intraoperative time was calculated from anterior pillar incision to complete hemostasis of the tonsillar fossa. Intraoperative blood loss was calculated by assessing the amount of blood absorbed by gauze (1 soaked 4 cm x 4 cm gauze = 10 mL) and the amount collected in the suction bottle. Postoperative pain at 24 hours and day 3 was evaluated using a Wong-Baker visual analog scale. In our study, we defined bleeding within 24 hours after tonsillectomy as reactionary hemorrhage requiring medical or surgical intervention and secondary hemorrhage as bleeding occurring within the 10th postoperative day.

Data were entered into MS Excel (Microsoft Corp., Redmond, WA), and a master chart was prepared. The analysis was done using SPSS 29. The data thus obtained was analyzed statistically and presented in the form of tables, figures, graphs, and diagrams where necessary.

## Results

The study population comprised a total of 350 patients in the age range of 10 years and above, consisting of 170 males and 180 females (Table 1). The mean duration of surgery was significantly longer for the cold steel dissection method, with a mean duration of 39.60 minutes, compared to the electrocautery technique, which had a mean duration of 14 minutes (P < 0.05). This significant difference in surgical duration between the two techniques was notable, with the electrocautery technique demonstrating a substantially shorter operative time.

**Table 1:** Demographic data of patients undergoing tonsillectomy using the cold steel dissection method and electrocautery technique

Gender	Cold Steel Dissection Method	Electrocautery Technique
Male	90	80
Female	85	95

Also, the cold steel dissection method had a significantly higher mean intraoperative blood loss (44.286 mL) than the electrocautery technique (18.829 mL) (P < 0.05). This finding suggests that the electrocautery technique may be linked to lesser bleeding during surgery, which could possibly mean better outcomes for patients.

**Figure 1:**
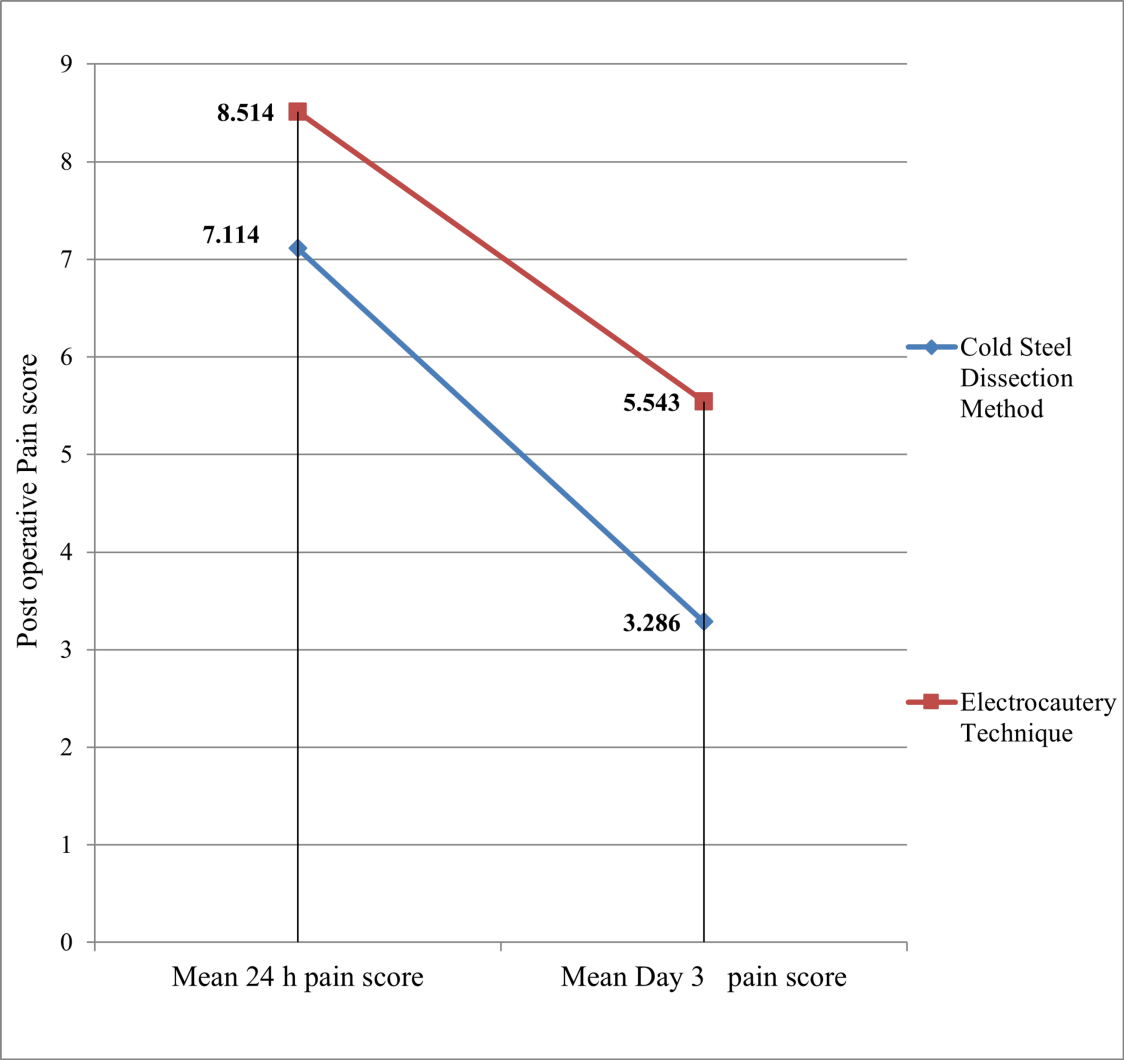
Comparison of postoperative mean pain scores between the cold steel dissection method and electrocautery technique

In terms of postoperative pain, the mean 24-hour postoperative pain score was significantly lower for the cold steel dissection method, with a mean score of 7.114, compared to the electrocautery technique, with a mean score of 8.514 (P < 0.05). The average pain score three days after surgery was also significantly lower for the cold steel dissection method (3.286) compared to the electrocautery technique (5.543) (P < 0.05) (Figure 1). These findings suggest that the cold steel dissection method may be associated with reduced postoperative pain.

Another uncommon problem in our study was bleeding after surgery, which happened only once in both the cold steel dissection and electrocautery groups. Table 2 summarizes the overall results of the study variables, offering a comprehensive overview of the findings. This study's results add to what are already reported about comparing cold steel dissection and electrocautery techniques in tonsillectomy by showing the pros and cons of each.

**Table 2:** Comparison of study parameters between the cold steel dissection method and electrocautery technique

Type of Tonsillectomy	Cold Steel Dissection Method			Electrocautery Technique		
Study parameters	Min	Max	Mean	Min	Max	Mean
Duration of surgery (in mins)	16	66	39.60	8	23	14
Intraoperative blood loss (mL)	16	63	44.29	11	29	18.83
24-hour postoperative pain score	6	9	7.11	7	9	8.51
Day 3 postoperative pain score	3	6	3.29	3	7	5.54

## Discussion

The surgical removal of tonsils, known as tonsillectomy, is a very common procedure in the field of otorhinolaryngology (ENT). The traditional method of tonsillectomy, known as cold steel dissection, has been widely used in the past. However, advancements in technology have led to the development and adoption of more modern techniques including electrocautery, laser dissection, harmonic scalpel, and coblation. Currently, though tonsillectomy procedures often employ advanced techniques, there is still a need for further research to determine whether these modern methods offer significant advantages over the traditional cold steel dissection approach.

The ultimate aim of all tonsillectomy procedures is to optimize patient outcomes by reducing perioperative bleeding, pain severity, and postoperative morbidities and to determine the most effective approach to achieve these goals [9]. With this backdrop, we compared the outcomes and complications in tonsillectomy using the cold dissection method and electrocautery technique. We carried out a prospective observational study in patients undergoing tonsillectomy using the cold steel dissection method and electrocautery technique admitted to the Department of Otorhinolaryngology at a tertiary care center.

In our study, the mean duration of surgery was 14 minutes in the electrocautery technique and 39.6 minutes in the cold steel dissection method, which showed a statistically significant difference on analysis (P < 0.05 via the t-score test). The cold steel dissection method took longer because it required the use of gauze pieces to pack the tonsillar fossae and control bleeding, as well as the ligation of significant bleeders, which added to the overall surgical time. This finding is consistent with studies conducted by Dadgarnia et al. [10], Lassaletta et al. [11], and Chettri et al. [12]. The aforementioned studies suggest that electrocautery may offer a time-efficient advantage in surgical procedures.

Mean intraoperative blood loss was 18.83 mL in the electrocautery technique and 44.29 mL in the cold steel dissection method, with a statistically significant difference (P< 0.05). Significantly less intraoperative blood loss was found in the electrocautery group compared to the cold steel dissection group, which was corroborative with the findings of research by Pang [13], Silveira et al. [14], Chaudhary et al. [15], and Nunez et al. [16]. This finding is particularly significant in pediatric cases, as excessive bleeding can lead to serious complications, including fatigue, mental and physical distress, and even unfavorable outcomes. Minimizing blood loss is crucial in tonsillectomy procedures, especially in children, to ensure their safety and well-being. According to Pang's study [13], bipolar diathermy dissection is a precise and controlled method that allows accurate identification and diathermy of blood vessels with little blood loss. According to the study by Nunez et al. [16], hot dissection tonsillectomy significantly reduced perioperative blood loss by half compared to cold dissection tonsillectomy.

Postoperative hemorrhage is a potentially fatal complication that occurs in around 10% of patients. Bleeding occurring within 24 hours after tonsillectomy is taken as reactionary hemorrhage that was severe enough for any medical or surgical interventions. Bleeding occurring between day 1 and day 10 after the operation was taken as secondary hemorrhage. In our study, one case of reactionary hemorrhage occurred for the cold steel dissection method, and one case of secondary hemorrhage occurred for tonsillectomy using the electrocautery technique. This finding was similar to that reported by Leinbach et al. [17]. They found that there were no significant differences between the rates of primary and secondary bleeding after surgery and that it did not matter what dissection or hemostasis technique was used. Additionally, other studies such as those by Kujawski et al. [18] and Lee et al. [19] reported low and statistically insignificant rates of postoperative hemorrhage, further supporting our conclusions. The choice of technique may not be the sole determining factor in postoperative bleeding outcomes, and other parameters might play a role in determining bleeding risk.

In our study, the mean 24-hour pain score was 8.51 in the electrocautery technique and 7.11 in the cold steel dissection method. The mean pain score on day 3 was 5.54 in the electrocautery technique and 3.29 in the cold steel dissection method. After the analysis, it was found that the P-value was <0.05, showing a statistically significant difference in both the 24-hour pain score and the day 3 pain score. Higher pain intensity scores with electrocautery in our results correspond with other studies including Silveira et al. [14], Leinbach et al. [17], and Mofatteh et al. [9]. A consistent finding across all studies was the significant increase in postoperative pain with electrocautery tonsillectomy, which is comparable to our study.

Our study has some limitations, such as a sample that was chosen at random and different surgical techniques used, but it still tells us a lot about how well these two techniques work and how we need to tailor surgical approaches to each patient to get the best care.

## Conclusions

Our study compared the outcomes of tonsillectomy using the cold steel dissection method and the electrocautery technique. The electrocautery technique offers shorter recovery times and less bleeding but slightly higher pain levels, whereas the cold steel dissection method is associated with less pain but longer recovery times and more bleeding. Notably, postoperative hemorrhage rates were similar between the two techniques. Our findings suggest that electrocautery may be suitable for patients with limited blood volume, offering reduced bleeding and faster recovery without increased hemorrhage risk, though with slightly more pain.
